# Glycopyrrolate does not influence the visual or motor-induced increase in regional cerebral perfusion

**DOI:** 10.3389/fphys.2014.00045

**Published:** 2014-02-11

**Authors:** Kim Z. Rokamp, Niels D. Olesen, Henrik B. W. Larsson, Adam E. Hansen, Thomas Seifert, Henning B. Nielsen, Niels H. Secher, Egill Rostrup

**Affiliations:** ^1^Department of Anaesthesia, RigshospitaletCopenhagen, Denmark; ^2^Functional Imaging Unit, Department of Diagnostics, Glostrup HospitalGlostrup, Denmark

**Keywords:** Cholinergic receptor antagonist, regional cerebral blood flow

## Abstract

Acetylcholine may contribute to the increase in regional cerebral blood flow (rCBF) during cerebral activation since glycopyrrolate, a potent inhibitor of acetylcholine, abolishes the exercise-induced increase in middle cerebral artery mean flow velocity. We tested the hypothesis that cholinergic vasodilatation is important for the increase in rCBF during cerebral activation. The subjects were 11 young healthy males at an age of 24 ± 3 years (mean ± *SD*). We used arterial spin labeling and blood oxygen level dependent (BOLD) functional magnetic resonance imaging (fMRI) to evaluate rCBF with and without intravenous glycopyrrolate during a handgrip motor task and visual stimulation. Glycopyrrolate increased heart rate from 56 ± 9 to 114 ± 14 beats/min (mean ± *SD*; *p* < 0.001), mean arterial pressure from 86 ± 8 to 92 ± 12 mmHg, and cardiac output from 5.6 ± 1.4 to 8.0 ± 1.7 l/min. Glycopyrrolate had, however, no effect on the arterial spin labeling or BOLD responses to the handgrip motor task or to visual stimulation. This study indicates that during a handgrip motor task and visual stimulation, the increase in rCBF is unaffected by blockade of acetylcholine receptors by glycopyrrolate. Further studies on the effect of glycopyrrolate on middle cerebral artery diameter are needed to evaluate the influence of glycopyrrolate on mean flow velocity during intense exercise.

## Introduction

Cerebral blood flow (CBF) is 50–60 ml 100 g^−1^ min^−1^ (Lassen, 1974) and its regional distribution is affected by cerebral neuronal activity and metabolism (Lassen, 1959). Thus, a motor task is followed by an increase in regional CBF (rCBF) (Olesen, 1971). Although regulation of activity-related CBF is not fully understood, it is considered to depend mainly on synaptic release of glutamate that releases a series of vaso-active substances from neurons and astrocytes (Attwell et al., 2010). However, other substances such as acetylcholine may be involved in maintaining CBF during, e.g., a change in perfusion pressure (Hamner et al., 2012). The endothelium is important for cerebral vasoregulation and acetylcholine interacts with endothelial muscarinic receptors (Tsukahara et al., 1986) to facilitate vasodilation (Faraci and Heistad, 1991). Intrinsic and extrinsic regulation of cerebral vessels and in turn CBF is described by Hamel (2006), and cerebral blood vessels receive cholinergic innervation originating mainly from the sphenopalatine ganglion and the nucleus basalis of Meynert (NBM) (Seylaz et al., 1988; Suzuki et al., 1990). The increase in rCBF during walking appears to include excitation of this NBM-originating cholinergic vasodilation system (Sato and Sato, 1995), suggesting a link between acetylcholine mediated control of vessels, and activity-dependent changes in perfusion. Similarly, the transcranial Doppler (TCD) ultrasound determined mean blood velocity for the contralateral middle cerebral artery (MCA *V*_mean_) increases during handgrip exercise (Jørgensen et al., 1993) and *V*_mean_ increases for the contralateral anterior cerebral artery (ACA) during movement of one foot while cycling exercise is associated with a bilateral increase in both MCA and ACA *V*_mean_ (Linkis et al., 1995). Activity of the cholinergic fibers induces increase in rCBF in dogs (D'Alecy and Rose, 1977; Toda et al., 2000) although there are conflicting findings as to the effect of atropine in blocking this dilation (D'Alecy and Rose, 1977; Busija and Heistad, 1981; Toda et al., 2000). However, there may be important differences among species. In swine the absence of change in perfusion to cortical areas of cerebrum during running (Delp et al., 2001) suggests that in quadrupeds running does not require higher brain activation. Accordingly, Hamner et al. (2012) suggest that cholinergic involvement in human CBF regulation needs to be directly addressed.

Acetylcholinesterase inhibitors that pass the blood brain barrier are used to reduce symptoms of Alzheimer's disease and vascular cognitive impairment (Birks and Harvey, 2006; Levine and Langa, 2011) possibly indicating a cholinergic contribution to regulation of CBF and cerebral metabolism. For other drugs crossing the blood brain barrier, such as atropine, the effect on CBF may be secondary to changes in cerebral metabolism. Glycopyrrolate is a similar muscarinic blocker that does not cross the blood brain barrier (Proakis and Harris, 1978) and during handgrip and cycling exercise, glycopyrrolate abolishes the exercise-induced increase in MCA *V*_mean_ (Seifert et al., 2010). This leads to the hypothesis that cholinergic vasodilatation is important for the motor task-induced increase in cerebral perfusion, not only during strenuous exercise, but also at a moderate activity level. We hypothesized that cholinergic vasodilation would induce an increase in rCBF upon cerebral activation. In order to test that hypothesis, we used arterial spin labeling (ASL) and blood oxygen level dependent (BOLD) functional magnetic resonance imaging (fMRI) to assess CBF with and without glycopyrrolate at rest and during a handgrip motor task and also during visual light stimulation.

## Methods

The subjects were 11 young healthy males at an age of 24 ± 3 years (height and weight 183 ± 6 cm, and 78 ± 9 kg; mean ± *SD*). The study was approved by the local ethical committee (J.nr. H-4-2010-066) and by the Danish Data Protection Agency (J.nr. 2011-41-6602) and written informed consent was obtained prior to participation in accordance with the Declaration of Helsinki. Inclusion criteria were male gender, being healthy non-smoker without taking any medication. The subjects abstained from strenuous physical activity on the day prior to the study and from caffeine on study days.

A catheter was inserted in an arm vein for administration of glycopyrrolate and also a catheter was placed in the radial artery of the non-dominant arm for blood sampling and cardiovascular measurements. Heart rate (HR) was monitored using a lead II ECG and arterial pressure was measured with a transducer (Edwards Life Sciences, Irvine, CA) at heart level and connected to a monitor (Dialogue-2000 IBC-Danica Electronic, Copenhagen, Denmark). Heart rate variability was quantified from data acquired at rest as the standard deviation of RR-interval duration divided by the mean duration. Mean arterial pressure (MAP), cardiac stroke volume (SV) and output (CO) were determined from the radial arterial pressure using pulse contour methodology (Nexfin, BMEYE B.V, Amsterdam, The Netherlands) (Bogert and Van Lieshout, 2005).

Magnetic resonance imaging was performed on a 3.0T Intera Achieva scanner (Philips Medical Systems, Best, The Netherlands) using an eight-element phased array receiver head coil. Structural imaging was performed using a 3D T1 weighted gradient echo sequence [repetition time (*TR*) = 10 ms, echo time (*TE*) = 5 ms, flip angle 8°, matrix 240 × 200, voxel size 1 × 1 × 1 mm, sensitivity encoding (SENSE) factor = 2]. The structural scan was used for tissue segmentation and calculation of brain volume.

Global brain perfusion was determined with velocity mapping using phase subtraction of a flow compensated and flow sensitive gradient echo sequence. Measurements were obtained with a matrix of 320 × 320 (*TR* = 12 ms, *TE* = 7 ms, flip angle 10°, voxel size 0.75 × 0.75 × 8 mm). The sequence was ECG gated (retrospective gating, 20 frames/cycle), using a velocity encoding of 150 cm/s. Any aliasing of the phase-difference was corrected using an in-house written routine for Matlab v 7.9 (The MathWorks Inc., Natick, MA). A fast angiography was performed and reconstructed in order for the imaging section to be positioned perpendicular to the cavernous segment of the internal carotid and basilar arteries.

Regional perfusion maps were obtained with ASL using the QUASAR sequence (Petersen et al., 2006). The sequence is based on multi-slice pulsed arterial labeling technique (EPISTAR) in which both the labeling and the control experiment are preceded by a saturation pulse and followed by a QUIPSS-II type saturation pulse. The readout was performed using a dual flip angle Look-Locker strategy and both crushed and non-crushed control-label pairs were acquired. General scan parameters were: *TR* = 4000 ms; *TE* = 22 ms, multiple time-point interval (Δ*TI*) = 300 ms, time of first readout (*TI*1) = 40 ms, flip angle 35/11.7°, matrix 80 × 80, voxel size 3 × 3 × 6 mm, gap 1.5 mm, SENSE factor = 2.5, 84 averages [48 at velocity encoding (*V*_enc_) = 4 cm/s, 24 without velocity encoding, 12 at low flip angle]. Seven transaxial perfusion slices parallel to the lower edges of the corpus callosum were acquired with a total scan duration of 6 min.

Functional imaging was carried out at rest and during task conditions using a standard gradient-echo EPI sequence with 32 slices positioned parallel to and overlapping the ASL acquisition. Variables were: *TR* = 3000 ms, *TE* = 35 ms, flip angle 90°, FOV 230 × 230 mm^2^, in plane resolution 2.9 × 2.9 mm^2^, slice thickness 4.0 mm, and inter slice gap 0.1 mm. For each run during task paradigms, 70 frames were obtained and for the resting state experiments, 300 frames were obtained.

The subjects were placed supine and run through a sequence of 2 times 30 min of scanning, the first being without influence of glycopyrrolate (Table 1). Measurements of global (velocity mapping) and regional (ASL) brain perfusion were performed with the subject at rest and while squeezing a rubber ball which required 36 N for 50% compression (Thera-Band® Hand Exerciser, prod. no. 26050, The Hygenic Corporation, 1245 Home Ave., Akron, OH 44310, USA). We instructed the subjects to compress the rubber ball by rhythmic squeezing ~30–60 times per minute with as much effort as possible, while lying still with the rest of the body.

**Table 1:** **The study protocol**.

**Time (min)**	**Baseline vs. glycopyrrolate**	**Scanning sequence**	**Arterial blood gas measurement**
00 min	Baseline	Survey/reference scan	
		Fast angio sequence	
		3D anatomy	
			1. PaCO_2_
		Global flow—rest	
		ASL—rest	
		Global flow—motor task	
		ASL—motor task	
		BOLD-fMRI—visual and motor task	
		BOLD-fMRI—motor task	
			2. PaCO_2_
50 min	Glycopyrrolate injection		
65 min	Glycopyrrolate	Survey/reference scan	
		Fast angio sequence	
			3. PaCO_2_
		Global flow—rest	
		ASL—rest	
		Global flow—motor task	
		ASL—motor task	
		BOLD-fMRI—visual and motor task	
		BOLD-fMRI—motor task	
110 min			4. PaCO_2_

*With the subjects placed in the scanner, two sets of measurements were obtained, the first (baseline) being without influence of glycopyrrolate. Global flow was obtained by velocity mapping in the carotid and basilar arteries, using phase subtraction. In addition, measurements included arterial spin labeling (ASL) and blood oxygen level dependent (BOLD) functional magnetic resonance imaging (fMRI)*.

Two runs of BOLD imaging were performed with different tasks, one in which the subject was stimulated visually with a checkerboard pattern reversing at 8 Hz. Furthermore, the subject was instructed to carry out sequential finger-thumb opposition on both hands while the checkerboard was on. In the other run, the subject squeezed the rubber ball intermittently with no visual stimulation. Both runs included 5 resting periods interleaved with 4 active, each lasting 30 s. Additionally, for assessment of intrinsic connectivity, BOLD data were acquired with the same variables, but for 900 s without any tasks imposed.

Arterial samples were obtained at rest in 8 subjects. The samples were emptied of any atmospheric content and immediately analyzed for carbon dioxide tension (PaCO_2_) (ABL 725, Radiometer, Copenhagen, Denmark), before and after the scanning sequence with and without glycopyrrolate. To induce cholinergic receptor blockade the subjects received 1.9 ± 0.34 mg of glycopyrrolate by stepwise infusions of 0.2 mg until no further increase in HR could be established.

### Region based fMRI analysis

Motor cortex (BA1) on the contralateral side of the activated dominant hand was drawn in the center of the anatomically defined region, around the area of maximal activation. Similarly, in the activated area in visual cortex (BA17) a central and extended area was delineated. The extended BA17 area corresponds to the entire region (left side) as defined the MNI/Juelich atlas. Data are derived from 22 measurements in 11 subjects.

### Statistical analysis

Variables were analyzed in STATA/SE v 11.2 (www.stata.com) using a random effect linear model and a *p*-value < 0.05 was considered statistically significant. Results are presented as mean ± *SD*. Functional BOLD data were analyzed using FSL v. 4.1.2 (FMRIB Software Library, www.fmrib.ox.ac.uk/fsl). Data were spatially aligned to correct for subject motion and slice timing differences and filtered with a temporal highpass filter matched to the paradigm period (60 s). Spatial filtering was with a 5 mm gaussian filter. Thresholding for positive and negative effects was done using a cluster based approach with a primary threshold of *z* > 2.3 and a cluster-level *p*-value of 0.05. The response magnitude was calculated and reported as average over all activation periods. Other data analyses were performed in Matlab.

## Results

### Cardiovascular variables

The cardiovascular effects of glycopyrrolate and the handgrip motor task are presented in Table 2. Glycopyrrolate increased HR from 56 ± 9 to 114 ± 14 beats/min (*p* < 0.001), diastolic (DIA) and MAP from 69 ± 7 to 82 ± 10 mmHg and from 86 ± 8 to 92 ± 12 mmHg, respectively, and CO from 5.6 ± 1.4 to 8.0 ± 1.7 l/min, while HR variability decreased from 5.0 ± 1.6 to 1.5 ± 0.4% (*P* < 0.001). In contrast, no effect was observed for systolic pressure (SYS). The handgrip motor task increased DIA and MAP by 4 and 5 mmHg, respectively (*P* < 0.001), but no significant changes were observed for SYS, HR, or CO. There was no interaction between glycopyrrolate and the handgrip motor task or visual stimulation on any of the cardiovascular variables. Also there was no significant difference between data obtained during the different MR measurements or the arterial blood gas samples (Table 3).

**Table 2:** **Cardiovascular variables**.

**Cardiovascular**	**Resting**	**Resting + glycopyrrolate**	**Motor task**	**Motor task + glycopyrrolate**
SYS (mmHg)	119±15	119±12^ns^	124±19^ns^	123±15^ns^
DIA (mmHg)	69±7	82±10^*^	72±8^ns^	84±8^**^
MAP (mmHg)	86±8	92±12^ns^	89±10^ns^	93±10^ns^
HR (B min^−1^)	56±9	114±14^**^	61±7^ns^	115±13^**^
SV (ml)	101±20	71±16^*^	101±22^ns^	72±14^*^
CO (l min^−1^)	5.6±1.4	8.0±1.7^*^	6.2±1.6^ns^	8.1±1.3^*^
CBF (ml/min)	797±162	817±203^ns^	856±162^ns^	837±204^ns^

SYS, DIA, MAP systolic, diastolic, and mean arterial pressure, respectively; HR, heart rate; SV, cardiac stroke volume; CO, cardiac output; CBF, cerebral blood flow. Values are mean ± SD. Testing performed with paired t-test vs. Resting (without glycopyrrolate). ns, non-significant;

* p < 0.05 and

** *p < 0.001*.

**Table 3:** **Arterial pH and carbon dioxide tension (PaCO_2_)**.

	**Rest**	**Resting + glycopyrrolate**	**Motor task**	**Motor task + glycopyrrolate**
pH	7.40±0.01	7.40±0.02	7.40±0.02	7.41±0.02
PaCO_2_ (kPa)	5.4±0.2	5.5±0.5	5.5±0.3	5.2±0.4

*Values are mean ± SD*.

### Whole brain flow velocity mapping by phase encoding

The total arterial flow to the brain was 801 ml/min, with an inter-subject SD of 156 ml/min (19.5%) and an intra-subject SD of 64 ml/min (8%) (Table 2). There was no significant effect of activation of handgrip or glycopyrrolate for the total or any of the three vessels [right and left internal carotid artery (ICA) or basilar artery]. The average maximal velocity for, e.g., the left ICA was 76 cm/s, with almost equal inter and intra-subject SD of 33 and 32 cm/s. For the right and the left ICA there was no significant effect of glycopyrrolate.

### Cerebral perfusion (arterial spin labeling)

Six subjects were evaluated with handgrip activation. Handgrip increased total gray matter CBF from 46 to 51 ml 100 mg^−1^ min^−1^ (*P* = 0.01), but the increase was not significantly altered by glycopyrrolate. Similarly, arterial spin labeling maps showed an area of significant flow increase corresponding to the left primary motor cortex during handgrip (*p* < 0.001, uncorrected), with no significant modulation during glycopyrrolate. However, a decrease in perfusion was seen in areas corresponding to the posterior cingulate and parietal cortices during glycopyrrolate (*p* < 0.001, uncorrected).

### Blood oxygen level dependent activation during handgrip

There was an increase of the BOLD signal during activation of the motor cortex (BA1) on the contra-lateral side of the activated dominant hand by 2.16% compared with the resting value (*P* < 0.001), but there was no effect of glycopyrrolate at voxel level.

### Blood oxygen level dependent activation during combined visual stimulation and motor task

**Figure 1:**
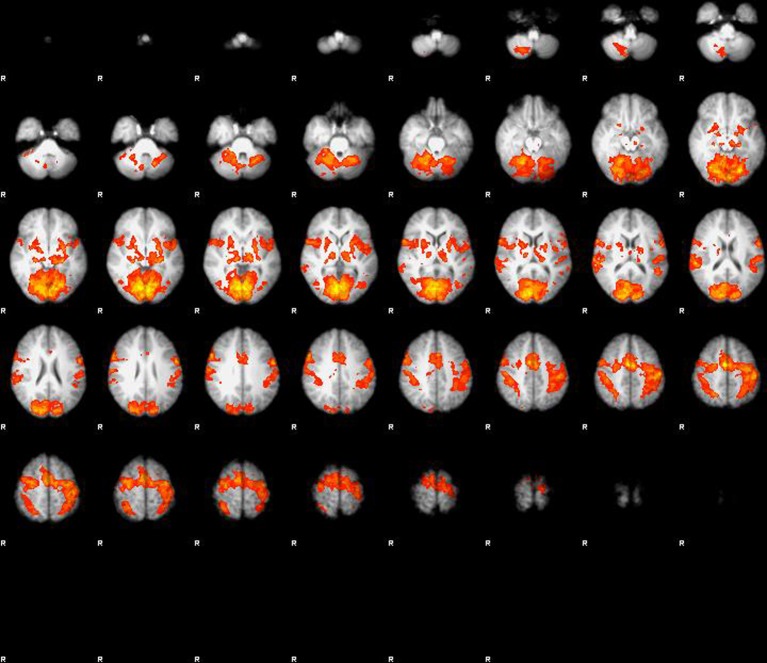
**Main effect of visuo-motor stimulation, large activation in extended areas centered around motor cortex (bilateral BA1), and visual cortex (BA17), but also including basal ganglia and cerebellum**. When properly corrected for multiple comparison or corrected voxel level (*p* < 0.05) there is no effect of glycopyrrolate.

The main effect of visuo-motor stimulation was a massive activation in extended areas centered around motor cortex (bilateral BA1), and visual cortex (BA17) and also including basal ganglia and cerebellum (Figure 1). In area BA1 on the contralateral side of the activated dominant hand, the BOLD response increased 1.9% during finger tapping and visual stimulation when compared to rest. In area (BA17) of the visual cortex the BOLD response increased by 2.76% in the central area and 1.16% in the extended area in response to visual stimulation and finger tapping when compared to rest (*P* < 0.001). No effect of glycopyrrolate was observed.

## Discussion

As expected, motor activation increased rCBF in motor cortex as indicated by BOLD and arterial spin labeling. The increase in rCBF did not affect total CBF as measured by phase mapping and apparently only whole body exercise increases total CBF. The dynamic behavior of CBF is supported by the increase in CBF during whole body exercise as detected by ^133^Xe clearance (Thomas et al., 1989) and by TCD (Jørgensen et al., 1992a,b), but in the present study we did not find any increase in CBF in spite of an increase in CO from 5.7 to 7.9 l/min during infusion of glycopyrrolate, nor an effect on rCBF during motor- and visual activation. Furthermore, we were not able to demonstrate that glycopyrrolate affected CBF increase during visual stimulation or a handgrip motor task.

Seifert et al. (2010) report that glycopyrrolate attenuated the increase in CBF during sustained handgrip and cycle exercise. There are several differences between the two studies, the most apparent being different modes of motor- and neural activation of the subjects and different methods of measuring brain blood velocity and flow. In our study handgrip increased MAP by about 5 mmHg, while no significant differences were induced in HR or CO, whereas, in comparison sustained handgrip in the study of Seifert et al. increased MAP, HR, and CO, 30 mmHg, 45 beats min^−1^ and 3.5 L min^−1^, respectively. Cerebral blood vessels are surrounded by nerve fibers that originate, respectively, from extrinsic peripheral nerve ganglia (sphenopalatine, otic, or trigeminal ganglion) and intrinsic brain neurons and contributes to the functional “neurovascular unit” where the vascular tone is regulated by both intrinsic and extrinsic innervation (Hamel, 2006). In our study the test persons may not be considered to perform “exercise,” but rather a light motor task and therefore, one can speculate that the intrinsic regulation is dominating an intravascular cholinergic stimulus. The study of Seifert et al. (2010) measured differences after strenuous exercise, including muscular pain, increased HR, CO, and respiratory frequency, where an extrinsic component might be of more importance. Since glycopyrrolate does not cross the blood brain barrier and therefore cannot affect the intrinsic regulation, a possible effect of blocking the cholinergic response might have been unobserved, or the cholinergic vasodilatation is only important under conditions where acetylcholine release from neuromuscular junctions influences total cerebrovascular reactivity, but not for the exercise-induced increase in regional cerebral perfusion during a handgrip motor task or visual stimulation.

The methods for measuring blood velocity and flow were different in the two studies. Seifert et al. (2010) used TCD of the middle cerebral artery. When measuring cerebral blood velocity using Q-flow, we considered that the small diameter of MCA would lead to an underestimate of flow because of a partial volume effect. We therefore, used the ICA although this measurement is directed toward total CBF compared to MCA where the motor cortex would represent a larger percentage of the distribution area. For measuring local cerebral blood perfusion we used ASL and BOLD. Being an indirect method of measuring cerebral perfusion, the BOLD technique measures changes in deoxyhemoglobin concentration that correlates to changes in local perfusion in the absence of metabolic changes, the cerebral tissue being uninfluenced by glycopyrrolate that does not cross the blood brain barrier. Importantly the CBF/perfusion baseline was not altered after administration of glycopyrrolate in neither study.

This study could be underpowered considering the increase in CBF during motor activity is about 60 ml/min during placebo and 20 ml/min during glycopyrrolate, but with the standard deviations above 150 ml/min, a very large sample would be needed in order to detect a potential effect of hand activity on global perfusion.

Although not a primary endpoint, we observed scattered areas of decreased perfusion during glycopyrrolate, especially in the posterior cingulate area. This finding is of potential interest, since posterior and parietal areas are affected early in the course of Alzheimer's disease in which cholinergic innervation may be deficient. A regionally specific effect of glycopyrrolate on brain perfusion has not been described and would need investigation in future studies.

## Limitations

The study is limited by the use of glycopyrrolate that does not cross the blood brain barrier and therefore, does not have any metabolic effect on cerebral tissue. Glycopyrrolate is therefore limited to influence the extrinsic innervation of the functional “neurovascular unit” leaving the intrinsic innervation unaffected. When performing a handgrip motor task or visual stimulation, the intrinsic innervation might be dominating and therefore mask the effect of cholinergic blockade.

Compressing an ergo ball rhythmically with the same force is not easily standardized and therefore, different levels of cerebral activation could bias the results, but there is no difference between handgrip task and light stimulation in interindividual cerebral blood perfusion response which supports the validity of the handgrip data.

The measurement of BOLD is an indirect measurement of cerebral perfusion as it determines changes in deoxyhemoglobin concentration that correlates to those in local perfusion in the absence of metabolic changes. The cerebral tissue being uninfluenced by glycopyrrolate, that do not cross the blood brain barrier, should therefore be without metabolic influence leaving the effect of cholinergic blockade to the extrinsic part of the neurovascular unit.

## Conclusion

Cerebral blood flow is not altered by glycopyrrolate during a motor task or visual stimulation as measured by fMRI. Further studies on the effect of glycopyrrolate on CBF comparing strenuous and light exercise using fMRI is needed to contribute to understanding the role of extrinsic vs. intrinsic cholinergic innervation on the regulation of CBF.

## Authors contributions

Kim Z. Rokamp participated in study design, collected the data, performed data analysis, and wrote the first draft of the paper. Niels D. Olesen collected the data and contributed to the preparation of the paper. Henrik B. W. Larsson, Adam E. Hansen, Thomas Seifert, Henning B. Nielsen, Niels H. Secher, Egill Rostrup participated in the study design, performed data analysis and contributed to the preparation of the paper.

### Conflict of interest statement

The authors declare that the research was conducted in the absence of any commercial or financial relationships that could be construed as a potential conflict of interest.
